# Prognostic Value of Pathological Personality Traits for Treatment Outcome in Anxiety and Depressive Disorders: The Leiden Routine Outcome Monitoring Study

**DOI:** 10.1097/NMD.0000000000001535

**Published:** 2022-04-23

**Authors:** Wessel A. van Eeden, Albert M. van Hemert, Erik J. Giltay, Philip Spinhoven, Edwin de Beurs, Ingrid V.E. Carlier

**Affiliations:** ∗ Department of Psychiatry, Leiden University Medical Centre (LUMC); † Clinical Psychology Unit, Institute of Psychology, Leiden University, Leiden, Zuid Holland, the Netherlands.

**Keywords:** Pathological personality traits, depression, anxiety disorders, treatment outcome, Dimensional Assessment of Personality Pathology–Short Form (DAPP-SF)

## Abstract

Previous studies have failed to take baseline severity into account when assessing the effects of pathological personality traits (PPT) on treatment outcome. This study assessed the prognostic value of PPT (Dimensional Assessment of Personality Pathology–Short Form) on treatment outcome (Brief Symptom Inventory [BSI-posttreatment]) among patients with depressive and/or anxiety disorders (*N* = 5689). Baseline symptom level (BSI-pretreatment) was taken into account as a mediator or moderator variable. Results showed significant effects of PPT on outcome, of which Emotional Dysregulation demonstrated the largest association (*β* = 0.43, *p* < 0.001). When including baseline BSI score as a mediator variable, a direct effect (*β* = 0.11, *p* < 0.001) remained approximately one-third of the total effect. The effects of Emotional Dysregulation (interaction effect *β* = 0.061, *p* < 0.001) and Inhibition (interaction effect *β* = 0.062, *p* < 0.001), but not Compulsivity or Dissocial Behavior, were moderated by the baseline symptom level. PPT predicts higher symptom levels, both before and after treatment, but yields relatively small direct effects on symptom decline when the effect of pretreatment severity is taken into account.

Pathological personality has often been linked to other psychiatric disorders, such as depressive and anxiety disorders (Bienvenu et al., 2001; Friborg et al., 2013; Shea et al., 2005). Personality pathology can be considered from a categorical as well as a dimensional perspective. From a categorical perspective, personality pathology is assumed to be present when a patient meets the criteria for a personality disorder according to the *Diagnostic and Statistical Manual of Mental Disorders* (*DSM-5*; American Psychiatric Association, 2013) or according to the Classification of Mental and Behavioral Disorders, 10th revision (American Psychiatric Association, 2013; First et al., 2015; World Health Organization, 1992). A meta-analysis demonstrated that the risk of comorbid personality disorders for major depressive disorder has been estimated at 45% (Friborg et al., 2014); the risk ranged from 35% to 52% for anxiety disorders (Friborg et al., 2013). Moreover, in multiple reviews and meta-analyses, researchers assessed the associations between personality disorders and treatment outcome of depressive and anxiety disorders (Feske et al., 1996; Kampman et al., 2008; Mulder, 2002; Newton-Howes et al., 2006, 2014; Olatunji et al., 2010; Reich, 2003). It was found that the odds for poor outcome more than doubled when a comorbid personality disorder was present (Newton-Howes et al., 2014). Evidence regarding anxiety disorders was less conclusive; some researchers found significant negative effects of personality disorder comorbidity (Feske et al., 1996; Reich, 2003), but others did not (Kampman et al., 2008; Reich, 2003). In one meta-analysis, Olatunji et al. (2010) found no significant effect of comorbid personality disorders on treatment outcome among patients with anxiety disorder.

There is clear empirical evidence that personality disorders are in fact better represented by a dimensional model than by the categorical model (Widiger and Samuel, 2005), in which personality pathology exists on a continuum, ranging from healthy/normal to maladaptive/abnormal psychopathology (Haslam et al., 2012). Several alternative dimensional approaches for personality disorders are proposed (see for an overview: Widiger and Simonsen, 2005). A major effort has been made in this regard by Livesley and colleagues, who reorganized lower-order traits described among 100 self-report scales into 18 factors (Livesley, 1998; Livesley et al., 1989). These 18 factors formed the basis for the development of a self-report scale—the Dimensional Assessment of Personality Pathology (DAPP; Livesley et al., 2008). Besides differences in methodology, subsequent studies found a considerable overlap with other models, such as with the five-factor model (Clark et al., 2002). The DAPP also demonstrated a considerable overlap in pathological personality traits (PPTs) with other relevant scales such as the Neuroticism-extraversion-openness (NEO) Personality Inventory (Clark et al., 2002), Personality Inventory for *DSM-5* (Gutiérrez et al., 2020; Van den Broeck et al., 2014), Schedule for Nonadaptive and Adaptive Personality (Pryor et al., 2009), and Severity Indices of Personality Functioning (Berghuis et al., 2014; Rossi et al., 2017). Moreover, the identified PPTs are often used as a proxy measure of the Alternative *DSM-5* model of personality disorders B-criterium personality traits (Berghuis et al., 2019).

Within the Leiden Routine Outcome Monitoring Study, it was demonstrated that patients with combined depressive and anxiety disorders displayed the highest mean values of PPT measured with the DAPP–Short Form (DAPP-SF), followed by patients with singular depressive disorders. Mean values of PPT were lowest for patients with singular anxiety disorders (Carlier et al., 2014). Van Noorden et al. (2012) and Schat et al. (2013) found that PPT predicted an unfavorable treatment outcome (50% reduction of measured psychological distress) in patients with mood, anxiety, and somatoform disorders, with a hazard ratio ranging from 0.92 (95% confidence interval [CI], 0.81–1.05) to 1.30 (95% CI, 1.12–1.51; Van Noorden et al., 2012). The present study builds upon this existing work with an extension of the sample, by using continuous outcome measures, and by explicitly taking the effects of baseline symptom level into account.

The effects of PPT on treatment outcome may be substantially lower when taking baseline symptom level into account, usually interpreted as severity. Baseline symptom level of depression and anxiety consistently influences posttest outcomes for depressive and anxiety disorders (Kampman et al., 2008; Mulder, 2002). The effect of PPT on treatment outcome or disorder persistence is attenuated when baseline symptom level is taken into account (Boschloo et al., 2014; Mulder, 2002; Spinhoven et al., 2011). For instance, the effects of neuroticism on the persistence of a depressive disorder over the course of 2 years decreased from 1.57 risk ratio (RR) (95% CI, 1.35–1.83) to 1.20 RR (0.92–1.57) and on the persistence of an anxiety disorder from 1.67 (1.42–1.95) to 1.09 (0.87–1.36), after adjusting for baseline symptom level (Spinhoven et al., 2011). Adjusting the relationship between PPT and treatment outcome for baseline severity may be too simplistic. After all, patients with high levels of PPT may report higher levels of depression and anxiety. Baseline severity may serve as a mediating factor between PPT and treatment outcome (Ananth et al., 2017). Candrian et al. (2007) investigated this and found that the effect of personality disorder on an 8-week open-label treatment of fluoxetine was fully mediated by baseline symptom level. Moreover, previous studies found differential clinical characteristics of high and low severe depression and anxiety (Batelaan et al., 2014; Kvarstein et al., 2013; Rhebergen et al., 2012; Wardenaar et al., 2014). Baseline symptom severity could be an important moderator of treatment outcome as is demonstrated for patients suffering substance use disorders (Ball et al., 2001) and borderline personality disorder (Bos et al., 2011; Sahin et al., 2018). Possibly PPT may be especially predictive for treatment outcome in patients suffering from higher baseline symptom levels. PPT may hamper coping with high disease severity of depression and anxiety (Pereira-Morales et al., 2018), in which case baseline severity could be a moderator variable of the effect of PPT on treatment outcome. Surprisingly, the likely intermediary effects (either as a mediator variable or a moderator variable) of baseline severity on the relationship between PPT and treatment outcome have received little attention in the current literature (Ball et al., 2001; Bos et al., 2011; Sahin et al., 2018).

Our aim was to investigate the prognostic value of dimensional PPT on treatment outcome among patients with anxiety disorders and/or depression while taking the effects of baseline symptom level into account. We first assessed the association between PPT and treatment outcome. Thereafter, we assessed how this possible association was affected by baseline severity. We assessed both the potential of mediation and moderation of baseline symptom level in the relationship between PPT and treatment outcome. The mediation analysis gave us an insight into the role of baseline severity within the relationship between PPT and treatment outcome. Moderation analysis gave us an insight into whether the effects of PPT on treatment outcome were different for patients with high baseline severity compared with those with low baseline severity. We used the DAPP-SF to measure a wide variety of maladaptive personality traits (van Kampen et al., 2008). Based on previous research (Boschloo et al., 2014; Mulder, 2002; van Eeden et al., 2019; Van Noorden et al., 2012), we hypothesized that PPT would be associated with higher symptom levels, both at baseline and after treatment. To assess the potential differential effects of PPT for depression, anxiety, and combined depression/anxiety (Carlier et al., 2014), we performed additional analyses for each diagnostic group separately.

## METHODS

### Participants

In this study, we used data from a sample of 5755 psychiatric outpatients who received treatment for anxiety and/or mood disorders at the mental health care provider GGZ Rivierduinen or at the Department of Psychiatry of the Leiden University Medical Centre (LUMC), both located in the Netherlands. We included adult patients (18 years or older) with anxiety disorders and/or depressive disorders of whom data were collected as part of the Leiden Routine Outcome Monitoring Study (2004–2013) and who had completed both the DAPP-SF at baseline and the Brief Symptom Inventory (BSI) at baseline and at 6 to 8 months posttreatment (see *Instruments*). Patients were recruited in policlinic departments for mood and/or anxiety disorder. When patients had other primary diagnoses, they were referred to other departments and therefore not included in the present study. As data collection in the form of Routing Outcome Monitoring is part of the routine care, this resulted in a representable sample of outpatients with anxiety disorders and/or depressive disorders.

### Design and Procedure

Routine outcome monitoring (ROM) data were derived from a prospective cohort study, which was carried out to assess treatment outcome for patients with mood, anxiety, and/or somatoform disorders in a naturalistic setting (de Beurs et al., 2011). For our analyses, we used data from assessments collected at the start of treatment and after 6 to 8 months of treatment. The first assessment occurred during an intake procedure; to diagnose patients in a standardized and reliable method, research nurses interviewed patients using the Mini International Neuropsychiatric Interview-Plus (Van Vliet et al., 2007). In addition, patients completed a number of self-report questionnaires. For further details regarding our ROM procedure, see de Beurs et al. (2011) and Carlier et al. (2018). Patients were treated in accordance with (inter)national evidence-based guidelines, consisting of pharmacotherapy, psychotherapy (e.g., cognitive behavioral therapy or interpersonal therapy), or a combination (*e.g.*, Cuijpers et al., 2013; van Fenema et al., 2012).

### Instruments

#### Pathological Personality Traits

The DAPP-SF is a 136-item self-report questionnaire used to assess maladaptive personality traits. Participants rated items on a 5-point scale, ranging from 1 (*very unlike me*) to 5 (*very like me*). The items are clustered into 18 subscales and four higher-order constructs. The subscales Submissiveness, Cognitive Distortion, Identity Problems, Affective Lability, Oppositionality, Anxiousness, Suspiciousness, Social Avoidance, Narcissism, Insecure Attachment, and Self-Harm are clustered under *Emotional Dysregulation* as the first higher-order construct with 78 items. The subscales Intimacy Problems and Restricted Expression are clustered under *Inhibition* as the second higher-order construct with 16 items. The subscales Stimulus Seeking, Callousness, Rejection, and Conduct Problems are clustered under *Dissocial Behavior* as the third higher-order construct with 34 items. Finally, the subscale Compulsivity equals the fourth higher-order construct *Compulsivity* with eight items (de Beurs et al., 2009).

In accordance with the DAPP-SF manual, subscale scores and higher-order construct scores are calculated as the mean of the item scores (see Table 1). Although the DAPP-SF subscales are associated with Cluster A, B, and C personality disorders, they can be considered as dimensional scales ranging from “normal” to maladaptive PPT. Psychometric evaluations, both in the community and in clinical samples (*i.e.*, patients with both axis I and axis II *DSM-IV* disorders), demonstrated good internal consistency, with Cronbach alpha between 0.78 and 0.89 (van Kampen et al., 2008). The DAPP-SF score ranges from 1 to 5 and was used in our study as the independent variable (IV), with the higher-order constructs serving as primary predictor variables.

**TABLE 1:** Demographic and Clinical Sample Characteristics at Baseline

Variable	Total Sample (*n* = 5689), Mean (SD) or *n* (%)
Age, y	38.8 (12.5)
Gender (female)	3572 (62.8)
BSI baseline score	1.33 (0.70)
BSI posttreatment score	0.85 (0.72)
MDD–single episode	1451 (25.5)
MDD–recurrent episode	2668 (46.9)
Dysthymia	682 (12.0)
Posttraumatic stress disorder	794 (13.6)
Social phobia	776 (8.5)
Generalized anxiety disorder	481 (8.5)
Panic disorder	1392 (24.5)
Obsessive-compulsive disorder	414 (7.3)
DAPP-SF (sub)scales	
Emotional Dysregulation	2.7 (0.66)
Submissiveness	2.9 (0.92)
Cognitive distortion	2.3 (0.95)
Identity problems	3.1 (0.99)
Affective lability	3.2 (0.85)
Oppositionality	2.8 (0.89)
Anxiousness	3.4 (0.92)
Suspiciousness	2.2 (0.98)
Social avoidance	3.0 (1.06)
Narcissism	2.4 (0.82)
Insecure attachment	2.9 (1.11)
Self-harm	1.8 (0.95)
Inhibition	2.8 (0.65)
Intimacy problems	2.4 (0.84)
Restricted expression	3.2 (0.85)
Compulsivity	2.9 (0.95)
Dissocial Behavior	1.9 (0.54)
Stimulus seeking	2.1 (0.81)
Callousness	1.8 (0.60)
Rejection	2.3 (0.82)
Conduct problems	1.4 (0.57)

DAPP-SF scales are demonstrated as mean item score (1–5).

MDD indicates major depressive disorder.

#### General Psychopathology

The BSI is a 53-item self-report questionnaire used to assess symptoms of depression, anxiety, somatization, obsessive-compulsivity, interpersonal sensitivity, hostility, phobic anxiety, paranoid ideation, and psychoticism (Derogatis et al., 1983). Participants rate items on a 5-point scale, ranging from 0 (*not at all*) to 4 (*extremely*). A psychometric evaluation of the BSI was performed in a large population of psychiatric patients, and it demonstrated good test-retest reliability and good internal consistency, with Cronbach alpha between 0.71 and 0.84 (De Beurs et al., 2006). The BSI score (total) ranges from 0 to 4 and was used in our study as a dependent variable (DV) for our statistical analyses.

### Statistical Analyses

**FIGURE 1:**
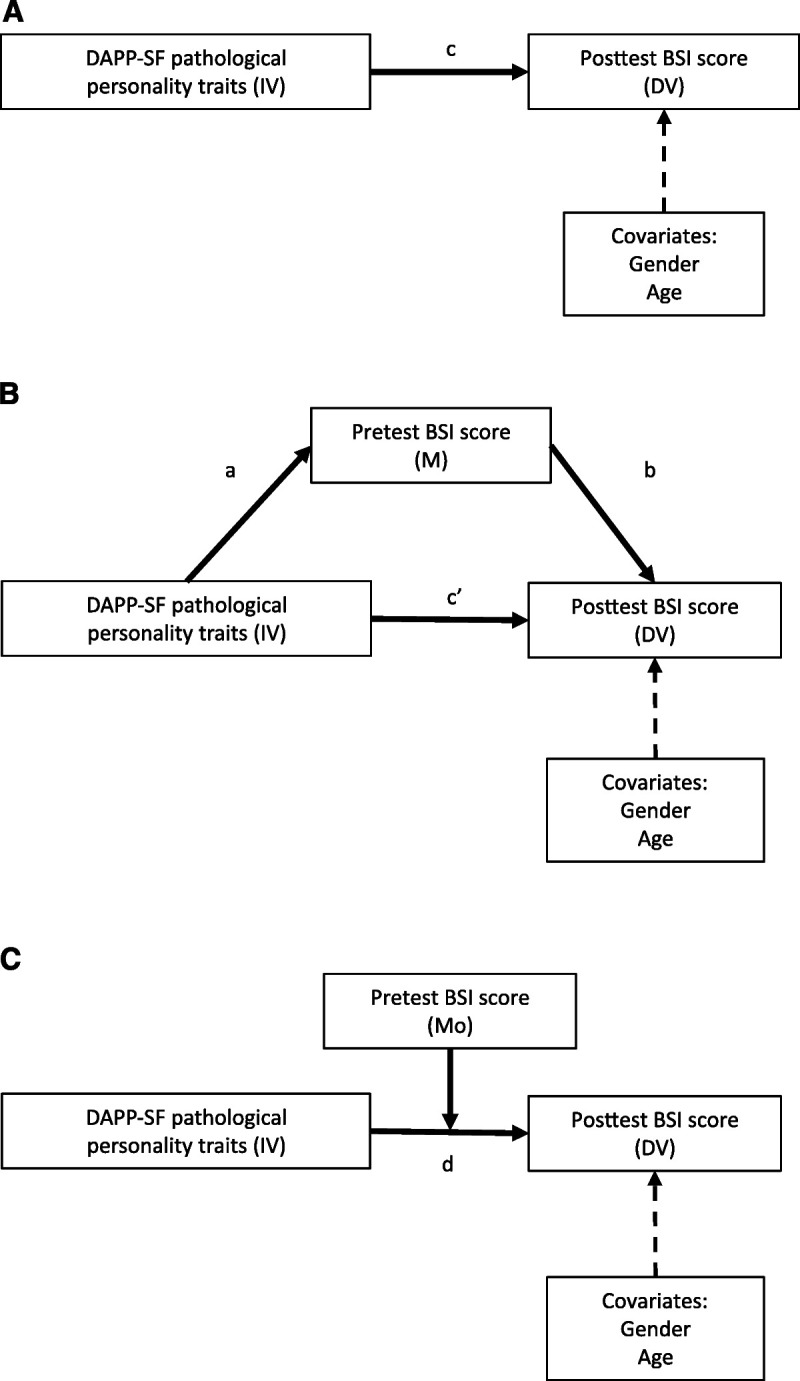
Model of psychopathology (DAPP-SF dimensions), baseline level of symptoms (baseline BSI score), and treatment outcome (posttest BSI score), suggesting that an increased baseline symptom level is an intermediate factor between psychopathology and treatment outcome. “IV” denotes independent variable (DAPP-SF). “DV” denotes dependent variable (posttest BSI score). “M” denotes mediating variable (baseline BSI). “Mo” denotes moderating variable (baseline BSI). “*c*” denotes the total effect of IV on DV. “*a*” denotes the effect of IV on M. “*b*” denotes the effect of M on DV. “*c*” denotes the direct effect of IV on DV. “d” denotes the moderated effect of IV on DV.

We took several steps in our analyses to investigate the prognostic value of dimensional levels of PPT and the intermediary effects of baseline symptom level on treatment outcome of patients with anxiety and depressive disorders. First, we conducted a mediation analysis using the Preacher et al. (2008) mediation model. This procedure allowed us to test the effects of an IV (higher-order PPT constructs) on BSI posttest (DV), either with or without a mediator (BSI baseline; M). This is demonstrated in Figure 1A, where the *c* path denotes the effect of PPT (IV) on treatment outcome (DV) without mediation by baseline symptom levels. Figure 1B demonstrates the *a* path, which denotes the effect of PPT (IV) on BSI (DV) at baseline (M), the *b* path denotes the effect of M on DV, and the c’ path denotes the direct effect after controlling for the mediator (M) baseline symptom level. Mediation was determined by testing the indirect effect of the IV on the DV via M (*a* × *b*). This is quantified as the product of the effect of the IV on M (*a* path) and the effect of M on the DV (*b* path). We used a bootstrapping approach with 5000 estimates of the *a* × *b* path to estimate the indirect effect. We computed 95% CIs for the empirical distribution, using cutoffs for the 2.5% highest and lowest scores. Mediating effects were considered to be significant when the CI did not include zero. For detailed information about the statistical procedures of the mediation analyses, see Hayes (2017) and Loose et al. (2018). Second, we performed a moderation analysis, in which PPT served as the IV, treatment outcome as the DV, and baseline symptom level as the moderation variable. We assessed whether there was an interaction between PPT and baseline symptom level in relation to treatment outcome. Thereafter, we assessed the effects of PPT for patients with 1 SD lower baseline symptom level and for patients with 1 SD higher baseline symptom level. We repeated these analyses for the 18 underlying DAPP-SF subscales clustered under the four higher-order constructs, and we performed additional analyses for each diagnostic group separately (depression, anxiety, or combined depression/anxiety groups), which is included in the Appendix (Supplemental Digital Content 1, http://links.lww.com/JNMD/A148). All outcomes and IVs were standardized (*i.e.*, *Z* scores) to yield standardized beta coefficients that could be compared between measures. Analyses were performed using R, version 3.4.1.

## RESULTS

### Sample Characteristics

Table 1 presents the sample characteristics. On average, patients were 38 years old (SD, 12.5 years), and women (62.8%) were overrepresented compared with men (37.2%). The mean (SD) BSI score was 1.33 (0.70) at baseline and 0.85 (0.72) after 6 to 8 months of treatment. The highest BSI scores were found among the combined depression and anxiety group (*p* < 0.001) (see Appendix Table 1, Supplemental Digital Content 1, http://links.lww.com/JNMD/A148). The DAPP-SF higher-order PPT constructs ranged from 1.90 (Dissocial Behavior) to 2.93 (Compulsivity). The highest levels of PPT were found among the combined subgroup compared with the depression and anxiety subgroups (see Appendix Table 1, Supplemental Digital Content 1, http://links.lww.com/JNMD/A148).

### Total Effect of PPT on Treatment Outcome

The total effect of PPT on treatment outcome is presented in Table 2, under “Total Effect of PPT (IV) on Treatment Outcome (DV)” (see also Fig. 1A). Table 2 shows the total effect of PPT on treatment outcome, which is defined as the posttreatment BSI score. All higher-order constructs of PPT were significantly associated with treatment outcome (*i.e.*, less improvement), ranging from *β* = 0.10 (SE = 0.02, *p* < 0.001) for Compulsivity to *β* = 0.43 (SE = 0.02, *p* < 0.001) for Emotional Dysregulation. We found similar results for the subgroups anxiety, depression, or combined group (see Appendix Table 2, Supplemental Digital Content 1, http://links.lww.com/JNMD/A148).

**TABLE 2:** Predicting Treatment Outcome With DAPP-SF Higher-Order Constructs of PPTs Mediated by Baseline Level of Symptoms Within Patients With Depression and/or an Anxiety Disorder (See Also Fig. 1A and B)

Independent Variable (IV)	Total Effect of PPT (IV) on Treatment Outcome (DV)	Direct Effect of PPT (IV) on Treatment Outcome (DV)	Effect of PPT (IV) on Baseline Symptom Level (M)	Effect of Baseline Symptom Level (M) on Treatment Outcome (DV)	Mediating Effect
*Denoted in* Figure 1 *as*	**c**	**c’**	**a**	**b**	**a × b; 95% CI**
Total (*n* = 5689)					
Emotional Dysregulation	0.43**	0.11**	0.67**	0.45**	0.31 (0.28–0.33)
Inhibition	0.24**	0.08**	0.32**	0.51**	0.17 (0.15–0.18)
Compulsivity	0.10**	−0.02	0.22**	0.54**	0.12 (0.11–0.14)
Dissocial Behavior	0.15**	0.04*	0.22**	0.53**	0.12 (0.10–0.13)

All variables are standardized. DAPP-SF subscale represents the independent variable (IV), baseline (BSI sum score at baseline) represents the mediating variable (M), and posttest (BSI sum score at follow up) represents the dependent variable (DV). “*c*” denotes direct effect, “*c*” denotes total effect, “*a*” denotes the effect of IV on M, “*b*” denotes the effect of M on Y, “*a × b*” denotes indirect mediating effect. Analyses are adjusted for age and gender.

*p < 0.01.

**p < 0.001.

**FIGURE 2:**
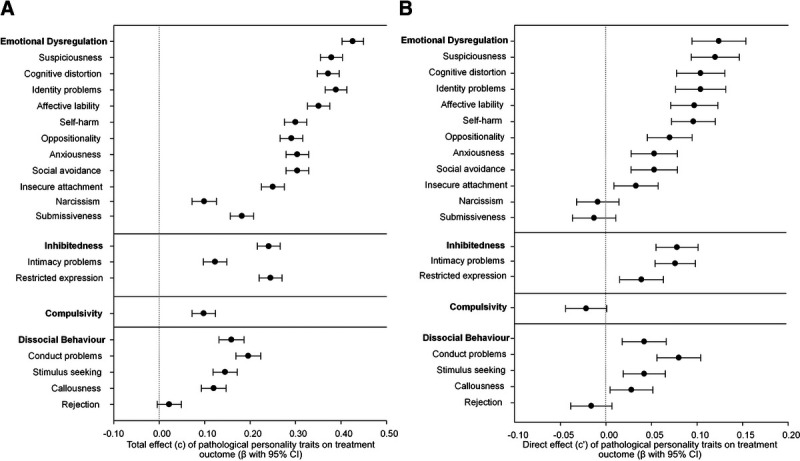
A, Total effect (c) of individual DAPP-SF PPTs on treatment outcome (posttest BSI score). B, Direct effect (c’) of individual DAPP-SF PPTs on treatment outcome (posttest BSI score).

Regarding the individual subscales underlying the higher-order constructs (Fig. 2A and Appendix Table 3, Supplemental Digital Content 1, http://links.lww.com/JNMD/A148), we found beta coefficients ranging from *β* = 0.02 (SE = 0.01, *p* = 0.09) for Rejection to *β* = 0.39 (SE = 0.01, *p* < 0.001) for Identity Problem. The subscales Identity Problems (*β* = 0.39, SE = 0.01, *p* < 0.001), Suspiciousness (*β* = 0.38, SE = 0.01, *p* < 0.001), Cognitive Distortion (*β* = 0.37, SE = 0.01, *p* < 0.001), and Affective Lability (*β* = 0.36, SE = 0.02, *p* < 0.001) demonstrated the strongest effects and were all part of the Emotional Dysregulation higher-order construct. The subscale Rejection (part of the Dissocial Behavior construct) demonstrated a remarkably lower effect on treatment outcome compared with the other subscales.

### Association Between PPT and Baseline Symptom Level

The relationships between the DAPP-SF higher-order constructs and BSI baseline symptom level for the total group are presented in Table 2 under “Effect of PPT (IV) on baseline symptom level (M)” (see also Fig. 1B). We found that all constructs were significantly, *p* < 0.001, related to baseline BSI symptom level, ranging from 0.22 (SE = 0.02, *p* < 0.001) for Dissocial Behavior to *β* = 0.67 (SE = 0.02, *p* < 0.001) for Emotional Dysregulation within the total sample. We found no consistent differences in the magnitude of this association between the subgroups (see Appendix Table 2, Supplemental Digital Content 1, http://links.lww.com/JNMD/A148).

When assessing the underlying DAPP subscales of the higher-order constructs, we found large differences in association with baseline symptom level. The subscales Identity Problems (*β* = 0.61, SE = 0.01, *p* < 0.001), Cognitive Distortion (*β* = 0.57, SE = 0.01, *p* < 0.001), Suspiciousness (*β* = 0.56, SE = 0.01, *p* < 0.001), and Affective Lability (*β* = 0.53, SE = 0.011, *p* < 0.001) demonstrated the strongest associations with baseline symptom level and were all part of the Emotional Dysregulation construct. The subscales Rejection (*β* = 0.08, SE = 0.01, *p* < 0.001; “Rejecting others”) and Intimacy Problems (*β* = 0.09, SE = 0.01, *p* < 0.001) demonstrated the lowest associations regarding baseline symptom level and were part of Dissocial Behavior and Inhibition, respectively (see Appendix Table 3, Supplemental Digital Content 1, http://links.lww.com/JNMD/A148).

### Mediation of Baseline Symptom Level

The relationship between PPT and treatment outcome was mediated by baseline symptom level (Fig. 1B). Table 2 under “Mediating effect” shows the results of the mediation analysis of PPT in relation to treatment outcome, with baseline symptom level as the M (mediator). We found a strong mediating effect (*a* × *b*) of baseline symptom level, with coefficients ranging from *β* = 0.12 (95% CI, 0.10–0.13) for Dissocial Behavior to *β* = 0.31 (0.28–0.33) for Emotional Dysregulation.

The direct effect of PPT (c’), which takes into account the mediating effect of pretreatment level of symptoms, was approximately one third of the total effect and remained significant for Emotional Dysregulation, Inhibition, and Dissocial Behavior but was no longer significant for Compulsivity. This suggests that the effect is largely, but not entirely, mediated through the effects of baseline symptom level. The direct effect ranged from *β* = −0.02 (SE = 0.02, *p* = 0.071) for Compulsivity to *β* = 0.11 (SE = 0.02, *p* < 0.001) for Emotional Dysregulation. Individual DAPP-SF subscales demonstrated similar proportions of the total effect being mediated through baseline symptom level (see Fig. 2B). The direct effect was no longer significant for the subscales Narcissism, Submissiveness, and Rejection. On average, Emotional Dysregulation demonstrated the strongest effect on treatment outcome. There were no consistent differences in the diagnostic subgroups (see Appendix Table 3, Supplemental Digital Content 1, http://links.lww.com/JNMD/A148).

### Moderation of Baseline Symptom Level

Baseline symptom level was examined as a moderator of the relationship between PPT and treatment outcome (Fig. 1C) and is demonstrated in Table 3. Baseline symptom level was a significant moderator of the relationship between Emotional Dysregulation and Inhibition and treatment outcome. Interaction effects between PPT and baseline symptom level were statistically significant for Emotional Dysregulation (*β* = 0.061, SE = 0.010, *p* < 0.001) and Inhibition (*β* = 0.062, SE = 0.062, *p* < 0.001). No significant interaction effect was found for Compulsivity and Dissocial Behavior. The standardized simple slope of Emotional Dysregulation for participants with 1 SD below the mean of baseline was 0.070, the standardized simple slope for participants with a mean level of baseline severity was 0.130, and the standardized simple slope for participants with 1 SD above mean baseline severity was 0.191. The standardized simple slope of Inhibition for participants with 1 SD below the mean of baseline was 0.012, the standardized simple slope for participants with a mean level of baseline severity was 0.043, and the standardized simple slope for participants with 1 SD above mean baseline severity was 0.135. Thus, Emotional Dysregulation and Inhibition were most predictive of high BSI score after treatment among participants with high baseline symptom level. These results were similar across separate diagnostic groups, although for the anxiety subgroup, the interaction between Inhibition and baseline symptom level was no longer statistical significant. The results for each diagnostic group separately are demonstrated in Appendix Table 5 (Supplemental Digital Content 1, http://links.lww.com/JNMD/A148).

**TABLE 3:** Moderating Effects of Baseline Level of Symptoms When Predicting Treatment Outcome With DAPP-SF Higher-Order Constructs of PPTs, Within Patients With Depression and/or an Anxiety Disorder (See Also Fig. 1C)

Treatment Outcome: Posttreatment BSI Score	Interaction PPT (IV) With Baseline Symptom Level (Mo)		Effect PPT (IV) for 1 SD Below Mean Baseline Level of Symptoms (Mo)		Effect PPT (IV) for Mean Baseline Level of Symptoms (Mo)		Effect PPT (IV) for 1 SD Above Mean Baseline Level of Symptoms (Mo)	
*Denoted in* Figure 1 *as*			d – Low Baseline Symptoms		d		d – High Baseline Symptoms	
Independent Variable (IV)	Beta (SE)	*p*	Beta (SE)	*p*	Beta (SE)	*p*	Beta (SE)	*p*
Total (*n* = 5689)								
Emotional Dysregulation	0.061 (0.010)	<0.001	0.070 (0.017)	<0.001	0.130 (0.015)	<0.001	0.191 (0.019)	<0.001
Inhibition	0.062 (0.010)	<0.001	0.012 (0.016)	0.464	0.043 (0.012)	<0.001	0.135 (0.015)	<0.001
Compulsivity	−0.009 (0.011)	0.378	−0.010 (0.016)	0.546	−0.019 (0.011)	0.096	−0.028 (0.015)	0.061
Dissocial Behavior	−0.012 (0.011)	<0.265	0.052 (0.018)	0.003	0.039 (0.012)	0.001	0.028 (0.015)	0.066

DAPP-SF subscale represents the independent variable (IV). Baseline BSI score represents the moderator variable (Mo). Beta denotes standardized regression coefficients. SE denotes standard error. Analyses are adjusted for age and gender.

All subscales that were part of Emotional Dysregulation and Inhibition and with the addition of Rejection demonstrated significant interaction effects (see Appendix Table 4, Supplemental Digital Content 1, http://links.lww.com/JNMD/A148). Interestingly, among patients with a high baseline symptom level, Narcissism had a beneficial effect on treatment outcome, although with a small effect size (*β* = −0.34, SE = 0.016, *p* = 0.032).

## DISCUSSION

We examined the effects of dimensional levels of PPT on treatment outcome after 6 to 8 months of treatment in a large sample of outpatients with depressive disorders, anxiety disorders, and combined depressive/anxiety disorders. The findings support our hypothesis that PPT is strongly related to higher symptom levels both before and after treatment, even when patients do not meet criteria for a personality disorder. Patients with 1 SD higher dimensional level of PPT had, on average, 0.20 to 0.43 SD higher levels of general psychopathology (BSI) after receiving treatment. At first glance, this suggests that dimensional levels of PPT had a significant and seemingly clinically relevant predictive effect on treatment outcome. However, when taking baseline symptom level into account, we found that patients with high symptom levels at baseline had substantially higher symptom levels after treatment regardless of PPT level. Baseline symptom level could be considered an important mediator of the relationship between PPT and treatment outcome. PPT was related to higher baseline symptom levels. The direct adverse effect (c’) of PPT on outcome when baseline symptom level was taken into account was approximately one third of the total. This direct effect was no longer significant for Compulsivity. Furthermore, we found that the baseline symptom level moderated the predictive effects of Emotional Dysregulation and Inhibition, which were slightly more predictive of treatment outcome among participants with a high baseline symptom level. However, the effect size of this interaction was small. We found a similar effect of PPT on treatment outcome among the three patient groups (see Appendix, Supplemental Digital Content 1, http://links.lww.com/JNMD/A148).

Our results replicate findings of previous studies in which PPT was found to have a negative impact on treatment outcome in patients with anxiety and depressive disorders (Goddard et al., 2015; Schat et al., 2013; Shea et al., 1992; Telch et al., 2011; van den Hout et al., 2006; Van Noorden et al., 2012). Many studies, however, did not factor in the importance of baseline symptom levels. Because baseline symptom levels proved to have a strong and consistent relation to treatment outcome in the present and in previous studies, it is plausible that PPT has less prognostic value when researchers adjust for baseline symptom levels (Kvarstein et al., 2013; Mulder, 2002). Previous studies have also found higher levels of symptomatology (both pretreatment and posttreatment) when PPT was present, but with a similar symptom decline during treatment (van Eeden et al., 2019). Studies that adjusted for baseline symptom levels found (at most) a small effect of PPT on treatment outcome for both depressive and anxiety disorders, or no effect (Blom et al., 2007; Kampman et al., 2008). In this regard, the findings of the current study are in line with previous literature. We approached baseline symptom level as a mediating variable in which PPT is related to higher symptom severity and perceived stress at baseline, which in turn leads to higher levels of symptoms after treatment (Candrian et al., 2007). Moreover, for the PPT constructs Emotional Dysregulation and Inhibition, baseline symptom level served as a moderator variable, in which PPT was more predictive for adverse treatment outcome when patients experienced high symptom severity. This is in line with previous literature that found that baseline symptom severity was a moderator for treatment outcome for substance use disorders (Ball et al., 2001) and borderline personality disorder (Bos et al., 2011; Sahin et al., 2018). The present study is the first to assess the moderating effects of baseline severity on treatment outcome among depression and anxiety patients.

Conventionally, the relationship between PPT and depression/anxiety may be considered as an etiological one, in which PPT causes higher symptom levels of psychopathology. Researchers have demonstrated that PPT can be a predictor for future psychopathology in response to life stress (Malouff et al., 2005). Furthermore, PPT can cause increased levels of distress because it contributes to problems in physical health, increased financial difficulties, dissolution of relationships, and other negative life outcomes (Lahey, 2009). In line with this, we found that PPT was associated with higher symptom levels of depression and anxiety at both pretreatment and posttreatment. In particular, we found that Suspiciousness, Cognitive Distortion, Identity Problems, and Affective Lability related strongly to symptom level before and after treatment; these constructs may be especially linked to maladaptive reactions to life events.

PP is generally thought to be present before depression and anxiety; however, Widiger (2011) posited the presence of a *pathoplastic* as well as a *spectrum* relationship in addition to an *etiological* one. A pathoplastic relationship would suggest that the presentation and expression of PPT and psychopathology (in this case depression and/or anxiety) would bidirectionally influence each other. Both PPT and depression/anxiety are considered impairments to how an individual thinks, feels, and behaves in relation to others. A priori PPT results in higher levels of impairment in these areas, resulting in higher levels of reported depression/anxiety, but high levels of psychopathology may also influence the reported level of PP. Patients who are very anxious or depressed may fail to provide accurate self-descriptions (Fava et al., 1994; Gunderson et al., 2003; Widiger, 2011). Although some may consider the above as self-report bias, others argue that PPT causes patients to respond to stress with (or relapse in) depression. Thus, self-reported levels of depression are considered accurate expressions of underlying PP. Subsequently, patients who report lower (depression) symptom levels after treatment may also display a decrease in levels of PPT (Costa Jr et al., 2005). In further support of a pathoplastic relationship, levels of reported PPT were substantially higher when patients were diagnosed with both a depression and an anxiety disorder and had a higher BSI baseline symptom level. Unfortunately, we measured PPT only at baseline and therefore cannot make statements about the posttreatment decrease of PPT alongside the decrease of depression and anxiety. Alternatively, our findings can be interpreted in terms of a spectrum relationship. PPT and depression/anxiety can be (partly) considered as manifestations of one and the same underlying common spectrum (Widiger, 2011). In support of a spectrum relationship, we found the strongest associations with the higher-order construct of Emotional Dysregulation, which has demonstrated overlap with depression and anxiety. Symptoms of anxiety and depression may lie in the same spectrum as Emotional Dysregulation. In our study, PPT was measured at the same time point as baseline symptom level. According to earlier findings (Blom et al., 2007) and the theory of the pathoplastic and spectrum relationships, PPT was likely influenced by an individual's current depressive or anxious state, which could have affected our mediation analyses.

Our findings could be valuable for clinical practice with regard to making prognosis. We found that baseline symptom level had far greater prognostic value compared with PPT measured with the DAPP-SF. The DAPP-SF, however, was still of added predictive value. Moreover, the DAPP-SF may provide relevant patient-specific information, which may be a focus for psychological therapy (Antony et al., 2020; Berghuis et al., 2019). With regard to treatment, we found that patients with high levels of PPT experience higher symptom levels after 6 to 8 months of treatment for depression and anxiety. The implications regarding treatment can be interpreted in several ways. One can argue that patients with concurrent high levels of PPT do benefit from a treatment that does not necessarily focus on personality pathology. An additional treatment aimed at PPT may be appropriate only for patients who remain symptomatic in spite of treatment. Moreover, it is likely that patients with higher levels of PPT simply need to be treated longer to achieve full remission in symptoms (Boer et al., 2019). However, one could also argue that patients with high PPT should be treated differently or more intensely, to achieve the same symptom level after 6 to 8 months of treatment as their lower PPT counterparts (Angstman et al., 2011). Both of these treatment options need further research and policymaking, in which clinical aspects and efficiency play a role (Donohue et al., 2007; Helmchen et al., 2000).

### Strengths and Limitations

The strengths of our study include its large sample size and the distinction of diagnostic groups of depression and anxiety. By collecting data in a naturalistic setting, we were able to analyze data from a clinical sample, which was representative of day-to-day patient care. We also measured PPT dimensionally, which is considered a strength in light of how PPT is currently conceptualized. Previous studies have consistently criticized categorical definitions of PPT (*i.e.*, personality disorders), and there is still no consensus on how to best classify patients with personality problems (Newton-Howes et al., 2014; Widiger and Simonsen, 2005). Dimensional levels of PPT do not equate to personality disorders, but there is evidence that PPT could be a reasonable proxy for the personality disorder diagnosis itself (Bernstein et al., 2007; Few et al., 2016; Katz et al., 2018; Widiger et al., 2012). Contrary to most studies, we assessed the intermediary effects of baseline symptom severity as both a mediator and a moderator in the prospective relation of PPT to treatment outcome.

Our findings should also be considered in light of their limitations. First, personality pathology is a broad concept, which could also include other definitions such as psychodynamic functioning, personality organization, coping styles, attachment constructs, and so on. Although the DAPP-SF is based on 18 empirically sound factors (Livesley, 1998; Livesley et al., 1989) and increasingly used as a proxy measure for the Alternative *DSM-5* model of personality disorders B-criterium personality traits (Berghuis et al., 2019), caution is warranted when generalizations are made to other realms of personality. Second, with the current study design, causality between PPT and baseline symptom level was assumed but could not be formally analyzed because both were measured at the same time point. Mediation analysis is fitting when the results are interpreted as a etiological relationship between PPT and depression/anxiety. As discussed, the reality may be more complex. Third, we limited the assessment of outcome to 6 to 8 months of treatment. Some patients did not complete their follow-up and were left out of the analysis, potentially introducing selection bias (Hoenders et al., 2014). Fourth, we lacked information regarding the type of treatment patients received (psychotherapy, medication, or both). This may be relevant because certain treatments may be better suited to patients with PPT than others (van Bronswijk et al., 2018). Fourth, patients with personality disorders as primary diagnoses were referred to other departments and therefore not included in the present study. Therefore, our sample might not have been representative of patients with the highest levels of PP. Lastly, PPT was measured only once, and not repeatedly. Earlier studies demonstrated that a decrease of (self-reported) PPT can occur after psychopathology is treated and has declined (Fava et al., 1994; Gunderson et al., 2003; Widiger, 2011).

## CONCLUSIONS

We expanded the way in which researchers can examine the prognostic value of PPT for treatment outcome in depressive and/or anxiety disorders. Our results showed that PPT had a negative effect on treatment for patients with anxiety and depressive disorders, of which the PPT constructs Emotional Dysregulation and Inhibition among participants with high baseline symptom level demonstrated the strongest effect. This effect was, to a large extent, mediated by baseline symptom levels. High PPT was related to higher symptom levels both before and after treatment, and the added (direct) effect of PPT on symptom decline after treatment was relatively small. Moreover, the effects of Emotional Dysregulation and Inhibition were also moderated and demonstrated to have a stronger effect on treatment outcome when patients experienced high baseline severity, although with a small effect size.
